# Water Uptake in an Anion Exchange Membrane Based on Polyamine: A First-Principles Study

**DOI:** 10.1021/acs.jpcb.2c04115

**Published:** 2022-09-19

**Authors:** Eleonora Tomasino, Binayak Mukherjee, Narges Ataollahi, Paolo Scardi

**Affiliations:** Department of Civil, Environmental and Mechanical Engineering, University of Trento, Via Mesiano, 77, 38123 Trento, Italy

## Abstract

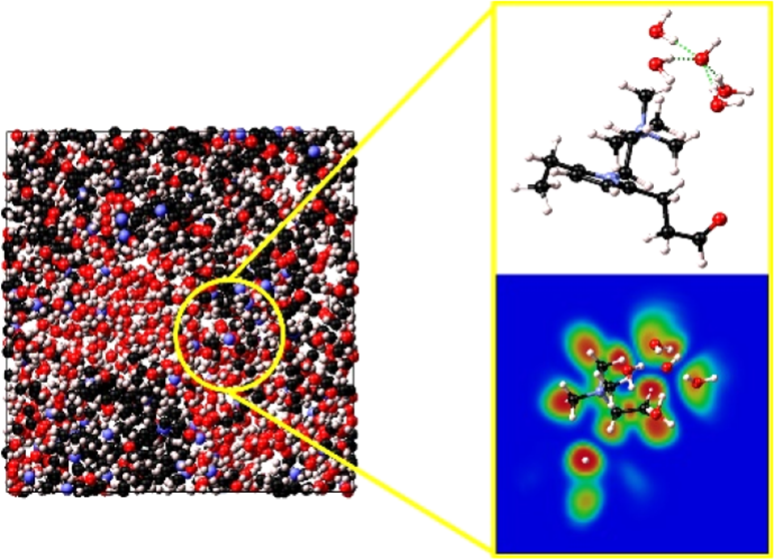

An atomistic level study of a single monomer of polyamine interacting with water molecules and hydroxide anions (OH^–^) was carried out to investigate the role of the polyamine structure in the hydrated morphology of anion exchange membranes (AEMs) for alkaline fuel cells and its influence on ionic conductivity and chemical stability. DFT calculations were performed to find the ground state of the system, studying the interactions of the solvent species with three different regions of the polymer—the amine functional group, the backbone, and the carbonyl group. The hydrophilic/hydrophobic behavior of each segment was determined, with calculated binding energies and Bader charge analysis providing a more quantitative analysis of the interactions and activation and reaction energies computed to investigate the chemical degradation mechanism. The results show the tendency of both OH^–^ and water molecules to form water clusters in the proximity of the ionized amine group. As such, these regions constitute the preferential pathway for ionic conductivity. Besides, the essential role of the water content is pointed out, not only to enhance conductivity but also to reduce degradation in an alkaline environment. The present work provides a baseline to assess the impact of polymer chemistry on the ionic conductivity of the membrane and acts as the first step for the development of high-performance AEMs and for an improvement of the overall performance of the fuel cell.

## Introduction

Ion exchange membranes consist of a polymer backbone (a rigid framework structure) and charged ionic groups which are covalently bonded either on the backbone or on the side chain. Depending on the ionic functional group, they can be classified into proton exchange membranes (PEMs) and anion exchange membranes (AEMs). PEMs contain negatively charged groups fixed to the backbone, which allow the transfer of cations (protons H^+^) but not of anions. Instead, AEMs have positively charged groups and enable the transfer of anions only (hydroxide ions OH^–^).^1^ PEM fuel cells (PEMFCs) and AEM fuel cells (AEMFCs), working at relatively low temperatures and high efficiency and overcoming the limits associated with the use of liquid electrolytes, have proved to be a promising technology for electric energy conversion.^1^

The use of polymeric membranes as solid electrolytes for fuel cells leads to several advantages, namely, higher power density, simplicity of construction, and the absence of leakage problems. Interest in AEMs has grown recently owing largely to the advantages of AEMFCs over PEMFCs—these include better kinetics for the oxygen reduction reaction, which gives rise to the opportunity of using non-precious metal catalysts and hence to a reduction of the device costs. In addition, corrosion problems can be minimized in an alkaline environment, and the AEMs are compatible with other fuels besides hydrogen, such as methanol.^2,3^ However, the low chemical and thermal stability in an alkaline environment and the lower ionic conductivity are still major issues to be solved. Optimized membranes should retain high ionic conductivity and at the same time good mechanical, chemical, and thermal stability under working conditions.

**Figure 1:**
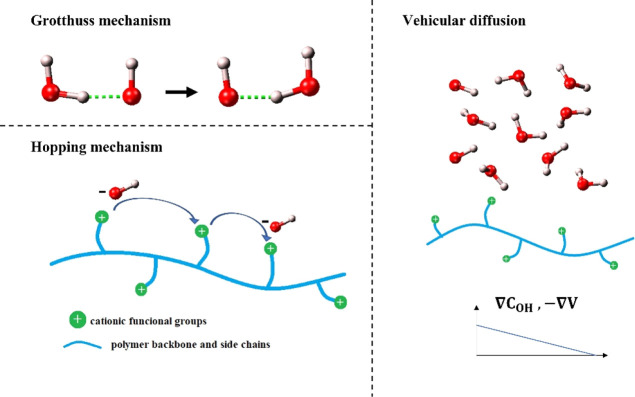
Hydroxide anion transport mechanisms in AEMs.

While the ion transport mechanisms in both AEMFCs and PEMFCs consist of a combination of the Grotthuss mechanism, vehicular diffusion, and hopping mechanism (Figure 1),^4−6^ there are some key differences between the two cases, concerning the role of the charged functional groups. The presence of cationic functional groups (e.g., trimethylammonium head groups) in AEMs inhibits the transport process since the partial positive charge acts as a trap for the hydroxide anions, neutralizing the ionic charge. In fact, the hydroxide, in alkaline conditions and at low water content, may react with the cationic groups, thus reducing the ion exchange capacity of the membrane, considering that only free ions contribute to the conductivity. Moreover, the degradation of the cationic groups leads to a decay of the performance and lifetime of the membrane itself.^7^ In this case, charge transport is more likely to occur in bulk water, that is, far from the backbone and side chains. In contrast, in PEMs, the anionic functional groups play an active role in promoting hydronium diffusion.^4,5^

The transport process in AEMs is strongly dependent on the level of hydration (λ) of the membrane itself (where λ is defined as the number of water molecules per OH^–^), on the water distribution and the solvation of the hydroxide anions.^8^ At low hydration levels (λ ≤ 3), when only the first solvation shell is present, the shielding effect of water is not enough to suppress the electrostatic interaction with the cationic group. At higher hydration levels (λ > 4), the vehicular diffusion mechanism is the dominant transport process of hydroxide anions in AEMs. It occurs when the hydrated hydroxide is hyper-coordinated to at least four electron-accepting water molecules, occupying the primary and secondary hydration shells, leading to the continuous formation and cleavage of hydrogen bonds in the water network, and it occurs in the presence of a concentration or electrical potential gradient. At high hydration levels, mixed Grotthuss and vehicular diffusion occur at high hydration levels, when the water structure is uniform and the water density is higher,^8,9^ whereas the hopping mechanism is negligible in all cases.^10,11^

The formation of water clusters and channels, necessary for ion transport, is strictly connected to the structure of the polymer and to the external conditions, and it will strongly affect the properties of the membrane. A proper hydration level must be ensured to achieve the optimum charge transport and prevent chemical degradation but at the same time, to avoid any problem related to membrane swelling. An excess of the water content, other than inadequate humidification, could cause the mechanical degradation of the membrane, thus nullifying the benefits obtained by an increase in conductivity.^12,13^

**Scheme 1:**
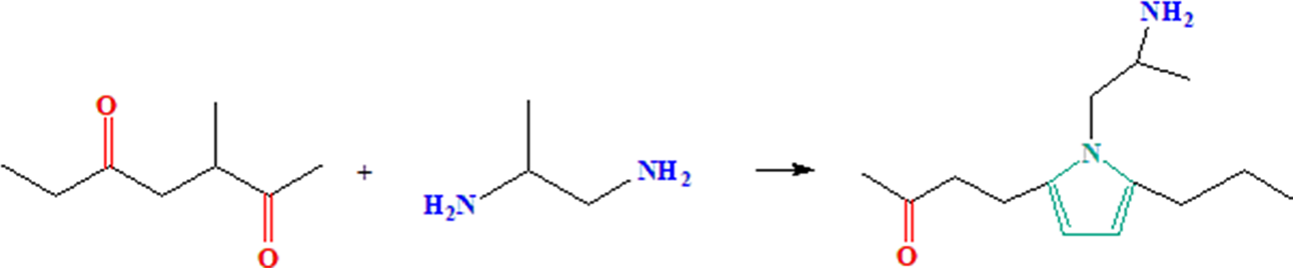
Polyamine Obtained From the Reaction of ter-polyketone with 1,2-Diaminopropane

**Scheme 2:**
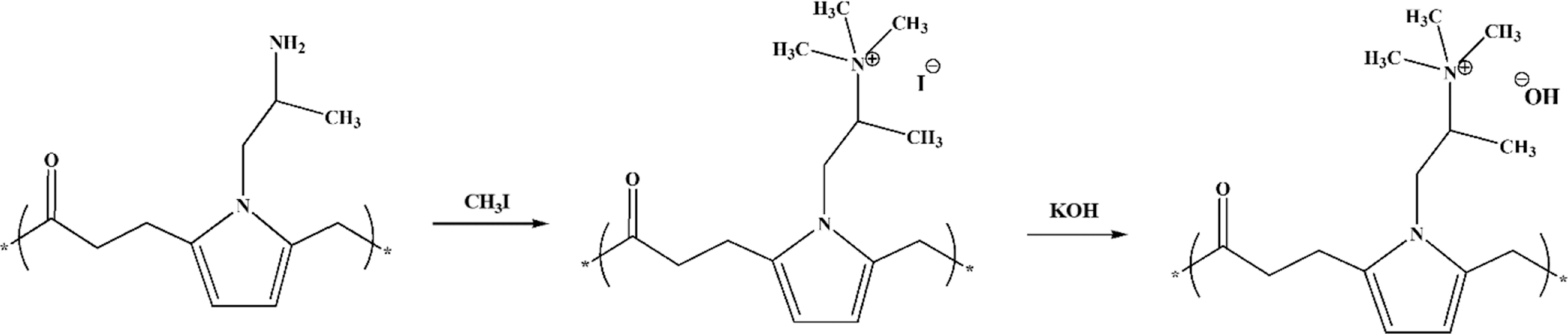
Methylation and OH-Exchange Reactions on Polyamine

AEMs based on polyamine can be obtained from ter-polyketone (ethylene, propylene, and carbon monoxide) and 1,2-diaminopropane through the Paal–Knorr condensation reaction, as shown in Scheme 1. The polyamine fragment consists of three different regions: a carbonyl group (C=O) directly connected to the backbone, the polymer backbone including a pyrrolic ring, providing rigidity and stability to the polyamine, and the amine functional group (NH_2_) connected to the pyrrolic ring through an alkyl chain. The latter is the active part of the polyamine, which is converted into a tri-methyl-ammonium (TMA) group after a methylation reaction with methyl iodide and subsequent OH-exchange (N(CH_3_)_3_^+^),^14−16^ (Scheme 2).

Each one of these regions has a different influence on membrane properties. A detailed analysis of each section separately can therefore shed light on the overall behavior of the membrane.

The complexity of the mechanism of water molecules and hydroxide ion transport through the AEM leads to the necessity of a detailed examination across different length- and time-scales. To this end, density functional theory (DFT) calculations are a useful first-principles technique, utilized extensively for the investigation of PEM properties.^4,17−20^ However, investigations on AEMs^6^ are less prevalent, and to the best of our knowledge, there have been no first-principles studies of AEMs based on polyamines.

In the present work, DFT simulations were carried out on a monomer of the polyamine (PA)^14−16,21−26^ to study the interactions of water molecules and hydroxide anions with a single monomer, eventually extrapolating information on the hydrophilic/hydrophobic nature of the polymer. In addition, the role of functional groups in ion transport and the influence of the hydration level on the degradation of the cationic group are analyzed. Electron localization functional (ELF) maps are used to study the nature of bonding and assess the presence of hydrogen bonds and the eventual formation of water clusters, as well as other kinds of interactions. Finally, binding energy, reaction energy, and activation energy calculations, together with Bader charge analysis, are performed to provide a more quantitative analysis of the interactions.

Extracting information from the atomistic level will enable the manipulation of the structure of the membrane on the nanoscale to eventually design optimum AEMs for alkaline fuel cell applications.^18^ Indeed, the degree of amination (conversion of carbonyl groups into pyrrole rings and segmental amines) of polyketone, which is achieved by varying the reaction times during the synthesis process of polyamine, is directly connected to the conductivity and to other membrane properties.

To this end, we investigated the interactions of the polyamine with water molecules, to define the hydrophilic/hydrophobic behavior of the different sections of the polymer, understanding the formation of water clusters and channels and hence to predict how the internal structure of the hydrated polyamine affects the overall properties of the AEMs. A complete knowledge of these factors is a necessary starting point for design and synthesis of high-performance AEMs.

## Computational Method

Molecular dynamics simulation for the initial relaxation of the polymer structure was carried out using the MedeA LAMMPS^27^ module of Materials design MedeA software. The job consisted of an initial *NVT* thermalization for 100 ps with a timestep of 0.5 fs at *T* = 293 K. The first relaxation process was followed by an annealing process carried out from 293 to 600 K and 1 atm, consisting in alternating 100 ps *NPT* ensembles and 50 ps *NVT* relaxation with temperature steps of 50 K and a timestep of 0.5 fs.^28−30^ Five annealing cycles were repeated until the density (1.06 g/mol) and the total energy (454.6 kJ/mol) reached stable values. All jobs were executed using the pcff+ force field^31^ and Nose–Hoover thermostat used for temperature control. The long-range Coulomb interactions were handled with the Ewald method with a default long range precision of 0.00001 and with a cutoff value of 9.5 Å. The long-range Van der Waals interactions were included via tail corrections.

Ab initio calculations based on DFT were performed using the VASP 6^32−34^ module of MedeA.^35^

Structure optimizations were carried out first on the “dry” (i.e., without water molecules) and “neutral” (considering non-ionized amine head group) isolated fragments using a conjugate gradient algorithm until the Hellman-Feynman forces on each atom were converged below 0.02 eV/Å, with a plane wave cutoff of 500 eV and real space projection. For all simulations, periodic boundary conditions have been applied, and the positions of the terminal carbons and hydrogens of the monomer backbone have been frozen, to account for the presence of neighboring monomers and adjacent chains present in the bulk system, which affect inter-molecular interactions and constrain the movement of the backbone. The initial optimization was performed using the Perdew–Burke–Ernzerhof (PBE)^36^ form of the generalized gradient approximation (GGA) of the exchange correlation functional, with Van der Waals DFT + D2 corrections due to Grimme.^37^ Subsequently, water molecules and hydroxide anions with both the ionized and non-ionized amine functional group were added to the initially optimized structure with different initial configurations, and the same structure optimization process was repeated.

Single point, self-consistent field (SCF) calculations were then performed on the minimized structures to determine the total and valence charge density (by means of Bader analysis), electron localization function, and activation and reaction energies. These simulations were performed using the more accurate Becke’s 3-parameter (B3LYP) hybrid exchange correlation functional, with Grimme Van der Waals corrections, an energy cutoff of 500 eV, and real space projection. The choice of the B3LYP functional relied on its greater accuracy and more efficient for the treatment of aqueous hydroxide ions.^18−20,38^ However, it is computationally more expensive and as such, has been employed only for single point analysis, while for structure optimization, the less demanding PBE functional was used.

Finally, the binding energies of solvent molecules were computed starting from the energies calculated for the optimized structures of dry systems, hydrated structures, and isolated water molecules and hydroxide anions. Besides DFT with the GGA-PBE exchange–correlation functional and Grimme Van der Waals interactions, the structures were optimized again with the optPBE-vdW functional of Klimeš et al.,^39^ a type of semi-local exchange–correlation functional^40,41^ that accounts for the Van der Waals dispersion interactions.

## Results and Discussion

### Selection of a Representative Monomer

**Figure 2:**
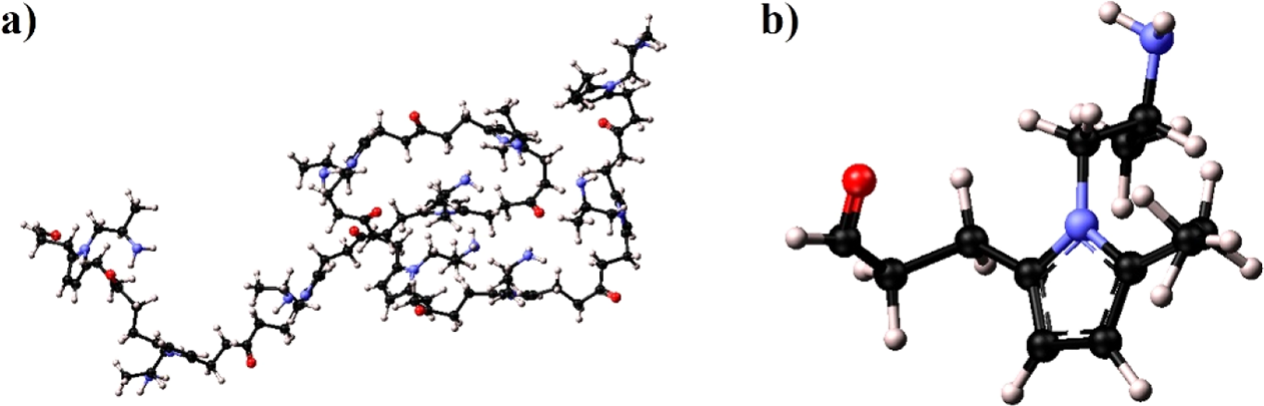
(a) PA polymer relaxed in MD simulation and (b) selected representative monomer.

The initial geometry, consisting of a PA polymer with 10 identical monomers (332 atoms) all containing a non-ionized functional group NH_2_, was built using the MedeA graphical interface. The initial structure was placed in a 50 × 50 × 50 simulation box with 3-D periodic boundary conditions but leaving enough vacuum space to avoid interactions between periodic copies. A simple LAMMPS MD thermalization at room temperature, followed by five annealing cycles up to 600 K, was executed to first optimize the polymer structure (Figure 2a).

**Figure 3:**
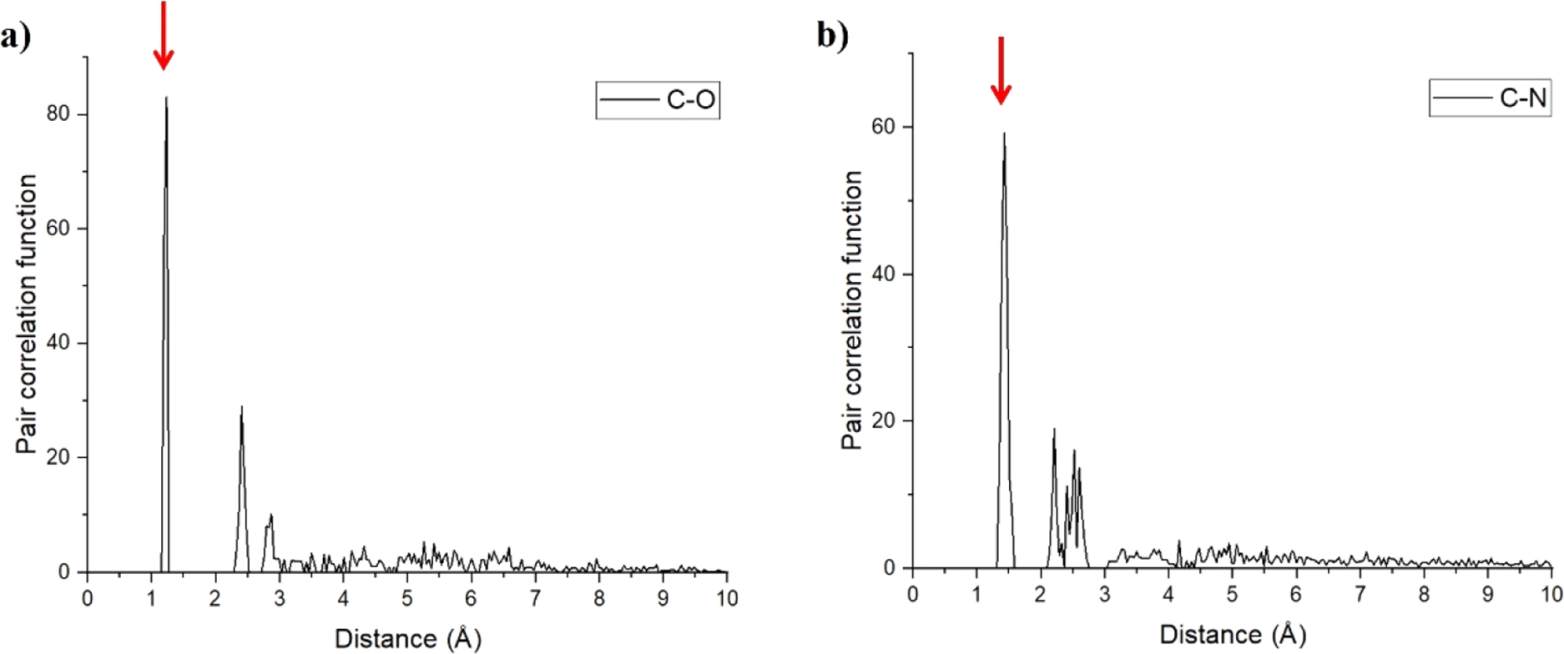
C=O (left) and C–N (right) pair-correlation analysis.

In order to check for structural variations between the different monomers, we performed a pair-correlation analysis, focusing on two representative bonds: C=O and N–C bonds in each carbonyl and an amine functional group in the polymer, respectively. As seen from the first peaks in Figure 3a,b, both these bonds present reasonably narrow and sharp distributions, indicating homogeneous bond-lengths across all monomers. As a further check, we tabulated all bond lengths and angles (Tables S1 and S2 in Supporting Information) of the N1 (nitrogen in the pyrrolic ring) and N2 (nitrogen in the functional group) atoms in each monomer, which we found to be quite similar. As such, we were able to confirm that a single monomer from the thermalized polymer could be used as a representative case to study its interaction with H_2_O and OH^–^ species.

### Structure Optimization and ELF Mapping

The selected monomer (Figure 2b) was subsequently used for the DFT simulations. Different initial configurations were simulated, with one water molecule or one hydroxide anion placed in different positions. The same analysis was performed on monomers with the ionized functional group (N(CH_3_)_3_^+^ head) interacting with one hydroxide anion and variable number of water molecules. Both ionized and non-ionized functional groups are in fact present in real membranes, the former obtained after the methylation reaction of the neutral membrane.

**Figure 4:**
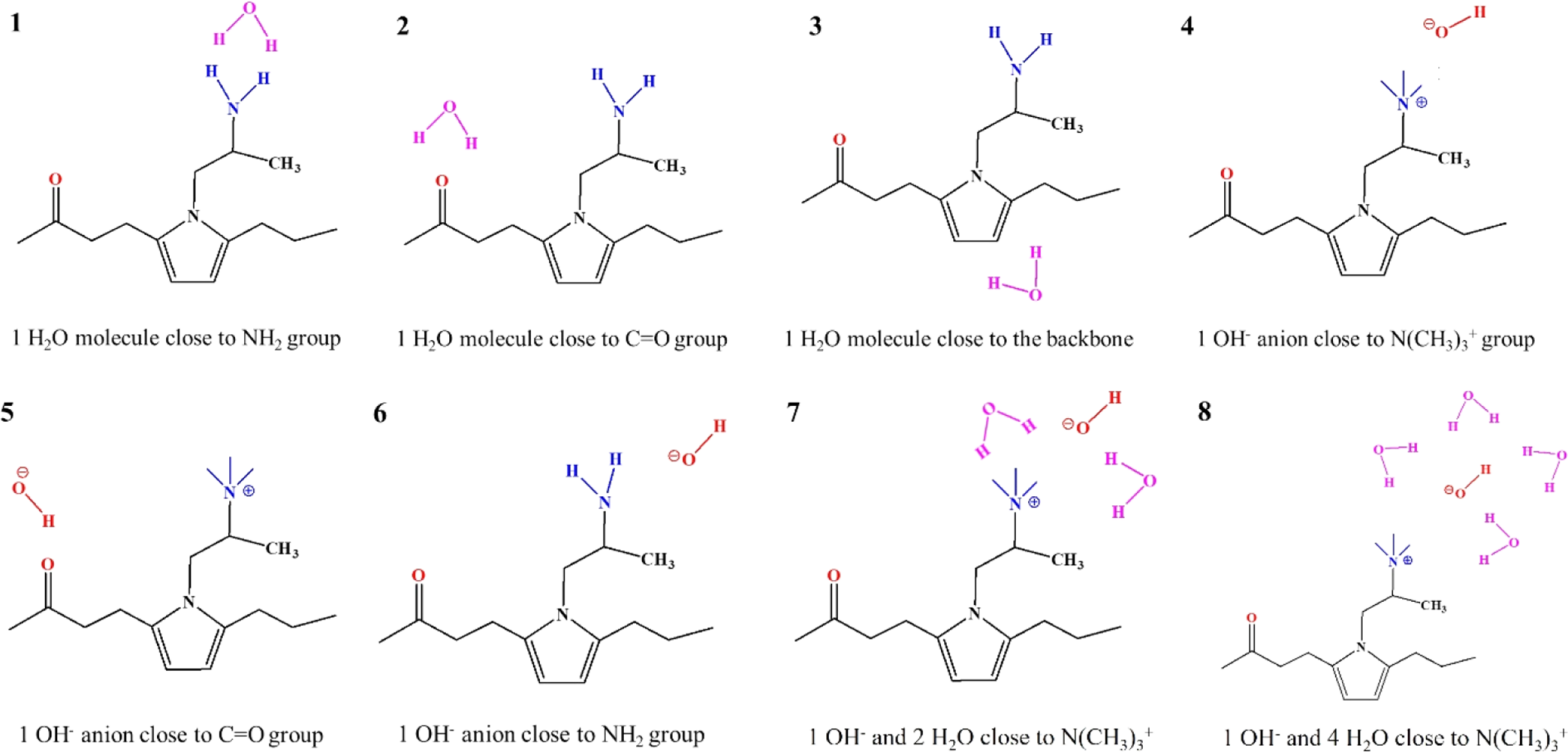
Initial configurations for DFT analysis.

The studied systems (Figure 4) can be loosely classified into three types: a single water molecule interacting separately with the NH_2_ and C=O functional groups and with the pyrrolic ring of the uncharged monomer (structures 1–3); a single OH^–^ ion interacting with the carbonyl and amine groups (structures 4–6), with the amine group now positively charged (N(CH_3_)_3_^+^); and finally, one OH^–^ and different numbers of water molecules interacting with the ionized functional group (structures 7, 8).

After a full structure optimization, the ELF was calculated to visualize the nature of the bonds formed during the minimization process. ELF maps are useful tools to distinguish between chemical bonding (shared-electron interactions, i.e., covalent and metal bonds) and physical bonding (i.e., ionic, hydrogen, and Van der Waals, with unshared-electron interactions) in simple molecular systems; moreover, the strength of the interaction depends on the ELF profile.^42^ The ELF profile is obtained by tracing the electron density around the interaction points or interfaces, and it is expressed as a dimensionless number n ranging between 0 and 1. A higher value of ELF, *n*(*r*) > 0.7, is representative of a stronger shared-electron interaction, where electrons are localized. Covalent bonds in molecules are detected, setting a value of *n* = 0.88. Instead, in hydrogen bonds, no shared electrons are localized in the regions between the molecules, but they can be visualized by the vicinity of their respective basins that sometimes are even touching, in the case of stronger hydrogen bonds, with n values around 0.3–0.2.^42^

**Figure 5:**
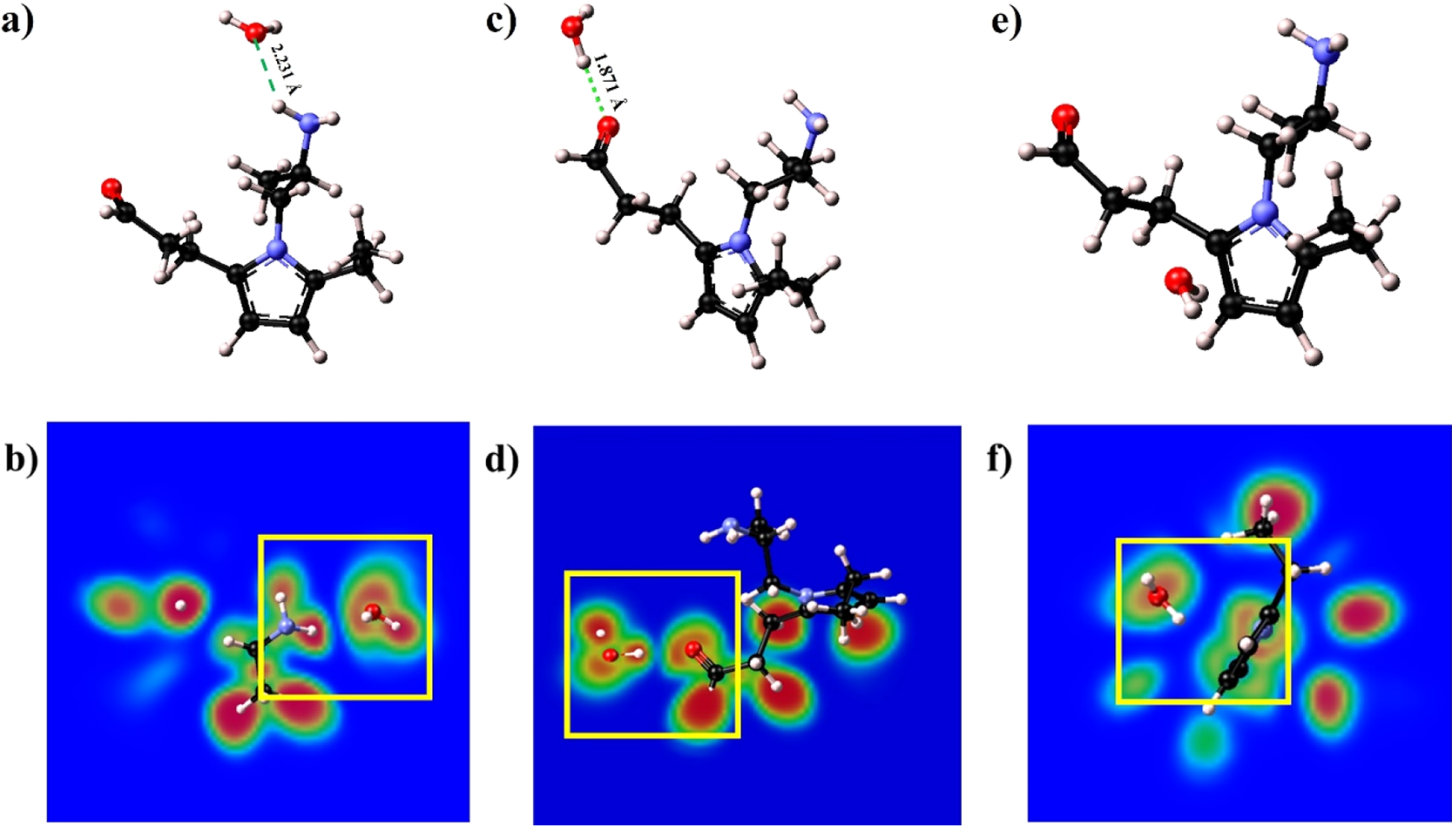
Optimized structures (a,c,e) and 2D ELF maps (b,d,f) of Structures 1, 2, and 3, respectively. The dashed green lines in (a,c) show the length of the formed bonds (2.231 and 1.871 Å).

The first three structures study the interaction of H_2_O with the different segments of the monomer. Optimized structure 1 is shown in Figure 5a. Here, the oxygen atom of the water molecule was oriented toward one hydrogen of the amine group, and stabilized at a distance of 2.231 Å. This configuration could suggest the presence of an interaction between the two atoms, which was subsequently established with the help of ELF analysis. Figure 5b depicts the ELF maps on a 2D plane intersecting the O atom of the water molecule and the closer hydrogen of the NH_2_ group (within the yellow square). In this, the surfaces are not touching, and they are not close enough to confirm the presence of a hydrogen bond involved. The shapes of the basins, though slightly deformed, are not perfectly round, which suggests the presence of a very weak interaction, possibly a weakly stabilizing dipole–ion interaction.^43^ It is possible to conclude that the presence of the alkyl chain is reducing the hydrophilic character of the amine group, thus preventing the formation of the hydrogen bond,^44^ and the non-ionized functional groups are only marginally contributing to the maintenance of membrane hydration.

As shown in structure 2 (Figure 5c), the water molecule formed a hydrogen bond with the oxygen of the carbonyl group, exhibiting a much shorter length compared to structure 1 (1.871 Å). This is a clue to a stronger hydrophilic behavior of the carbonyl group. Figure 5d shows the ELF map of structure 2, with the yellow square highlighting the ELF surfaces on the 2D plane of the carbonyl group and the closer hydrogen in the water molecule. This time, the presence of a hydrogen bond is clear since the two basins are touching, and the shapes are highly deformed. This indicates the presence of a strong H-bond between the two atoms.

Structure 3 (Figure 5e) was found to display a hydrophobic behavior. The water molecule, initially placed at circa 2 Å from the nitrogen of the pyrrolic ring, was then kicked away from the backbone. As shown in the pair correlation analysis (Figure S3), the distribution of N–O distances all lies above 3 Å, confirming the hydrophobicity of the backbone.

Subsequently, we studied the interaction of the OH^–^ ion with the head group and the carbonyl group. The hydroxide counterion is introduced by means of an OH-exchange process, by soaking the membrane into KOH solution.^14^ Depending on the degree of ionization, some ionized functional groups (with TMA head) will be present in the polymer along with the non-ionized ones; hence, they must be studied as well, having a leading role in the ionic transport mechanism.

The TMA functional groups are subjected to different degradation mechanisms at high temperatures and alkaline environments, mainly to nucleophilic substitution (S_N_2), Ylide formation, and Hofmann elimination.^25,26,45−47^ The degradation process is closely related to the level of hydration. Therefore, the interaction of the TMA head group with OH^–^ and different hydration levels will be analyzed with structures 4, 7, and 8, with λ equal to 0, 2, and 4 respectively, simulating the hydrated condition by explicitly adding water molecules to the system.

Besides, the interaction of the anionic species with the carbonyl group (structure 5) is simulated to investigate the chance of degradation of the polymer backbone in alkaline conditions and even with the non-ionized amine group (structure 6).

**Figure 6:**
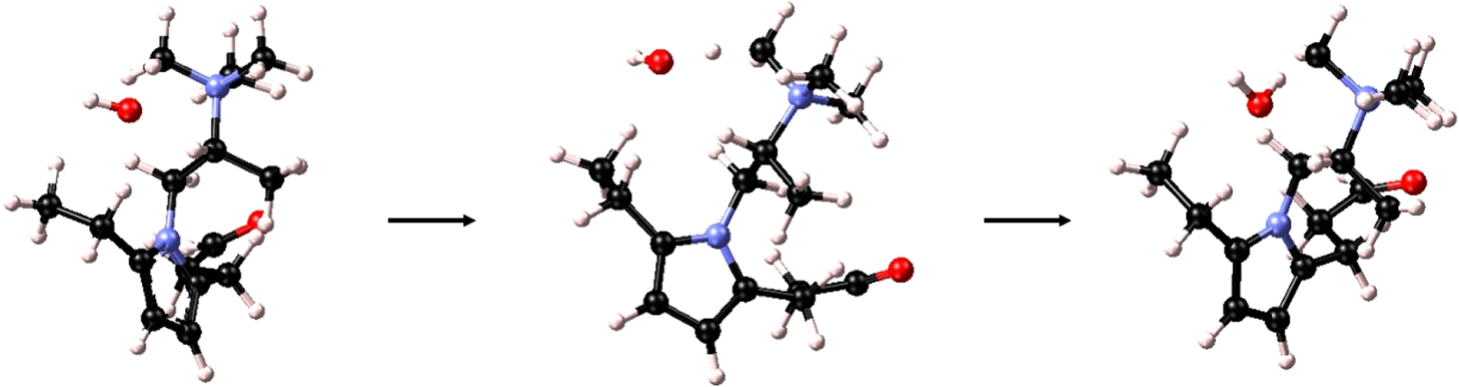
Optimization process and TMA head group degradation via Ylide formation of structure 4

From the DFT simulation of Structure 4 (Figure 6), it emerges that the OH^–^ tends to “steal” one hydrogen from the α-H in a methyl group of the TMA, causing the degradation of the cationic group. The Ylide formation degradation process is observed, with the formation of a water molecule and a N(CH_3_)_2_CH_2_ group. This kind of reaction is reversible and typically does not result in the degradation of the cationic group;^48^ however, it could be the starting point for further degradation reactions, such as the elimination of the entire methyl group.^49,50^

In this system, a S_N_2 elimination reaction was expected as well, with the attack of an α-C and the separation of one methyl group. However, this degradation process was not observed with the simulation, probably, because the activation energy of this reaction is higher than that for Ylide formation, which instead becomes the preferential pathway for degradation.

Besides, the reaction is strongly affected by the steric hindrance effect. Because of the bulky morphology of the monomer chain, the hydroxide anion is not always able to attack the α-H.

For the same reason, Hofmann elimination was not observed. This mechanism occurs with the attack of α-H in the β-position to the N atom (Scheme S1). Still, the steric interference caused by the vicinity of the β-H to the TMA group (2.355 Å) and the backbone (2.656 or 2.472 Å) is preventing any possible attack from the anionic species.

**Figure 7:**
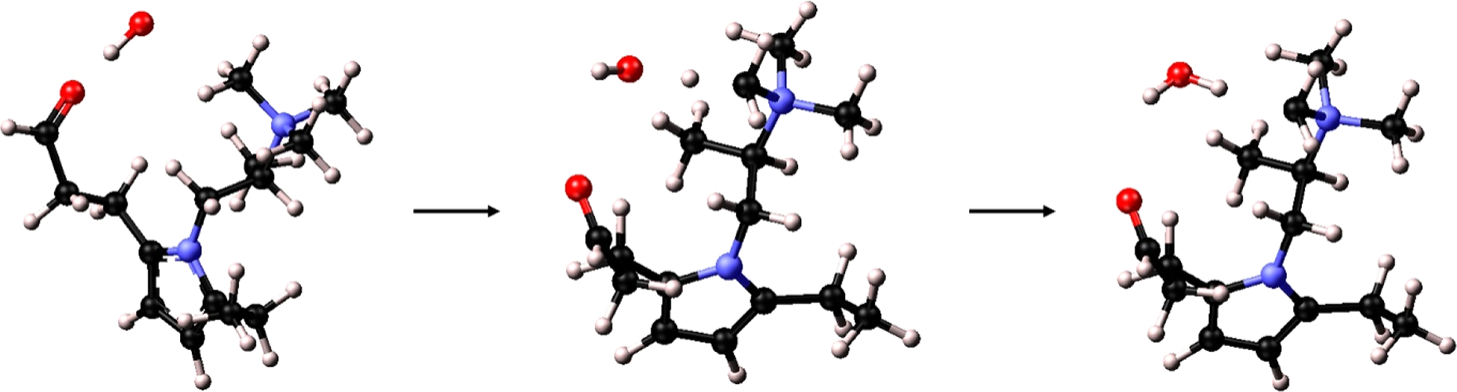
Optimization process and TMA head group degradation via Ylide formation of structure 5

Structure 5 (Figure 7) shows the interaction between the hydroxide anion and the carbonyl group. This time, the ion was repelled from the C=O group and then attracted by N(CH_3_)_3_^+^, forming a water molecule with the same degradation reaction of structure 4. This repulsive response can be justified by the presence of the two lone pairs of electrons in the latter, interacting with the negative charge of OH^–^. Based on this, we conclude that the hydroxide anions preferentially aggregate around amine groups only.

**Figure 8:**
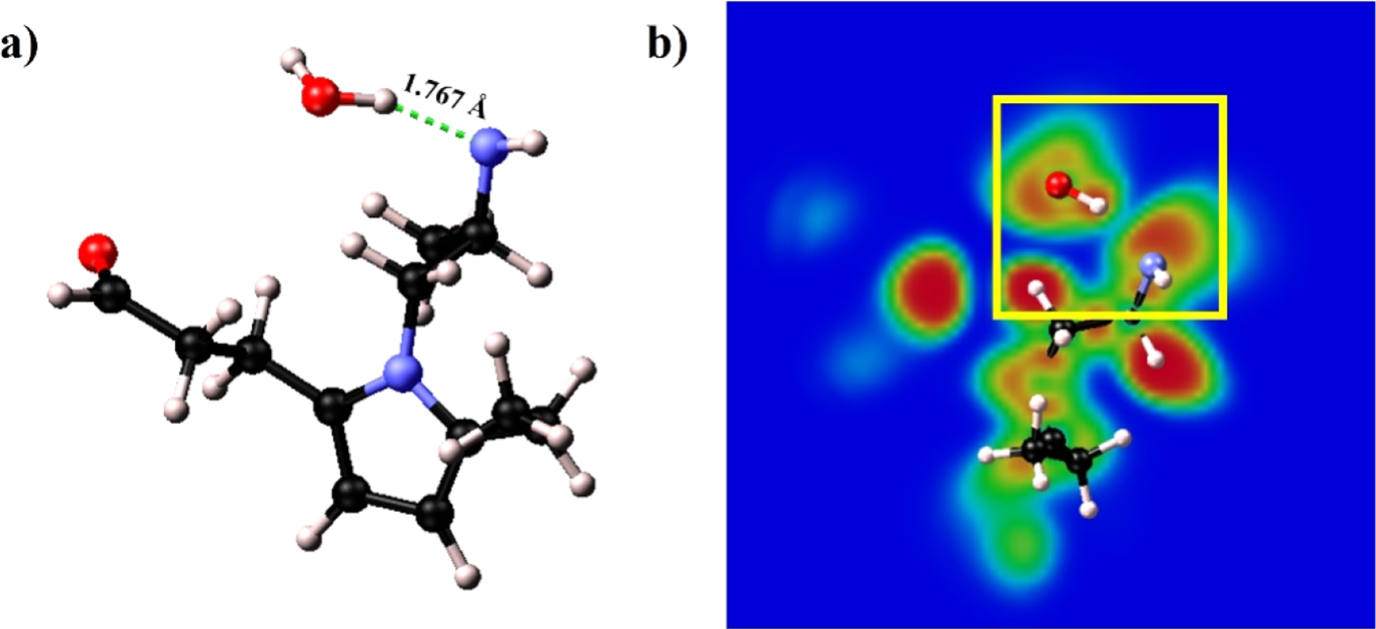
Optimized structures (a) and 2D ELF maps (b) of Structure 6. The dashed green lines in (a) show the length of the formed hydrogen bond (1.767 Å).

In structure 6, the hydroxide anion interacts directly with the non-ionized NH_2_ group (Figure 8a). The anionic species captures one hydrogen atom from the amine group, deprotonating the tertiary amine and then forming a hydrogen bond with it. Without water molecules, the NH_2_ group behaves as a stronger trap for hydroxide ions. The hydrogen bond length is shorter (1.767 Å), denoting the formation of a stronger bond compared to the interaction with the TMA group. However, this interaction is unlikely to occur since hydroxide anions are generally not present alone but surrounded by water molecules even at low hydration conditions, and the presence of water molecules in the proximity of non-ionized functional groups is not favored, given the much stronger hydrophilic behavior of carbonyl and ionized amine groups.

Finally, the last two structures aim at investigating how the degradation mechanism is affected by different hydration conditions, namely, at low hydration (λ = 2) and higher hydration levels (λ = 4). It is important to analyze both conditions, given that the water content is variable inside the membrane, approaching minimum levels (and even dry conditions) in the proximity of the cathode (where water is a reactant), and at high current density.^51^

**Figure 9:**
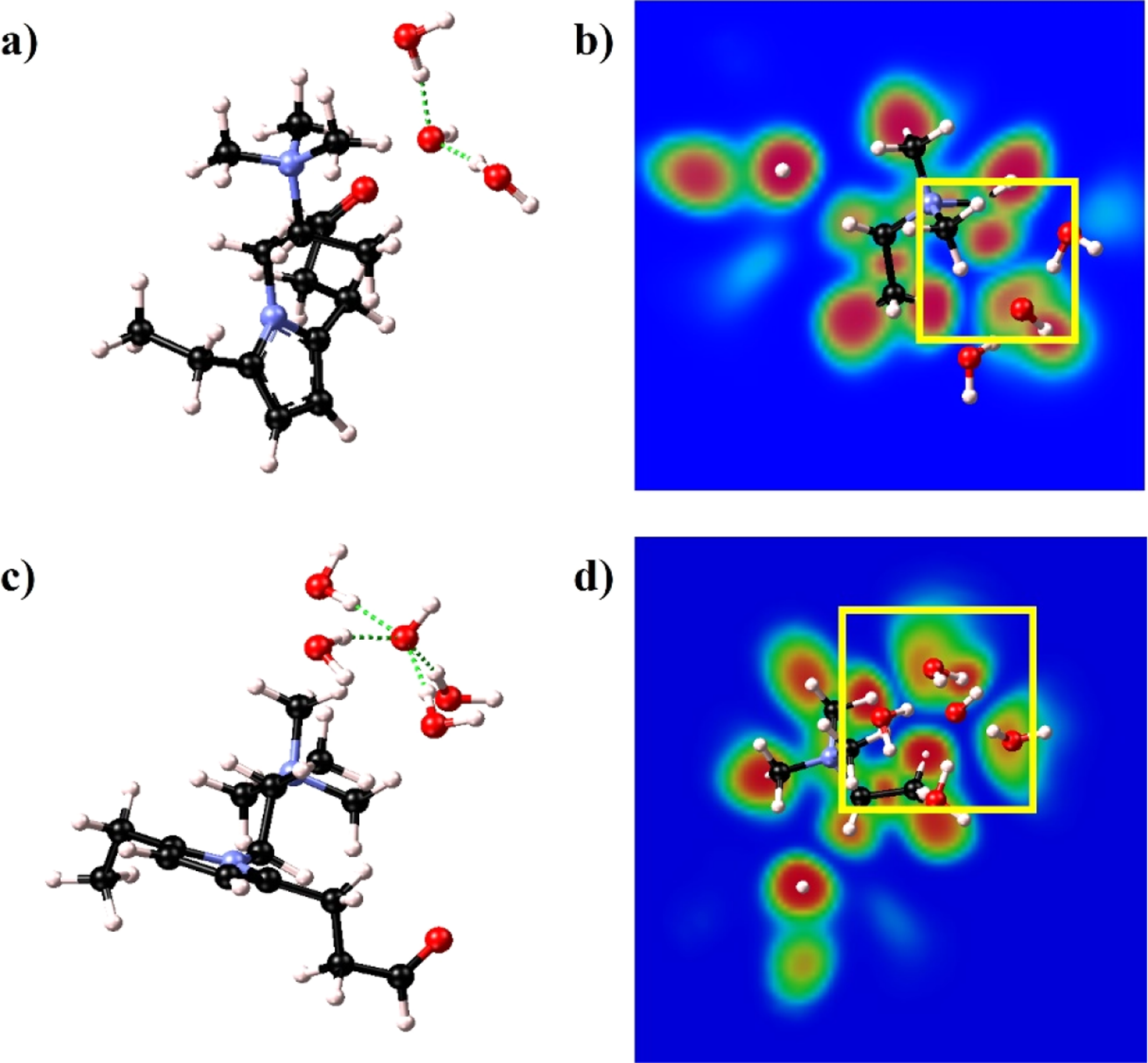
Optimized structures (a,c) and 2D ELF maps (b,d) of structures 7 and 8, respectively. The dashed green lines in (a,c) highlight the formation of the hydrogen bond chain and the solvation of the hydroxide anion.

In structure 7 (Figure 9a), the hydroxide anion interacting with the ionized head group is solvated by two water molecules in the first hydration shell, denoting low hydration conditions (λ = 2).

The 2D ELF map (Figure 9b) indicates the presence of a weak bond between the hydroxide and the TMA group, together with the hydrogen bonds formed with the water molecules. This is a clue for the formation of a small water cluster in the proximity of the ionized side chain.

Structure 8 (Figure 9c) simulates higher hydration conditions (λ = 4), with four water molecules in the first solvation shell of the OH^–^. With a completely solvated hydroxide anion, no interaction was detected with the head group, but rather the water molecules were arranged in such a way to shield the TMA, preventing any degradation and forming a chain of hydrogen bonds and weak interaction with methyl groups (Figure 9d), which again denotes the formation of water clusters in the proximity of the side chain.

Based on the preceding simulations, we can reasonably assert that the backbone and pyrrolic ring retain a hydrophobic behavior, while both carbonyl and amine groups are hydrophilic. The carbonyl group has a role in water uptake since it shows the strongest interaction with water molecules, but it does not have any active role in ionic conductivity, whereas the main path for hydroxide anions consists of the water clusters formed in the proximity of the ionized amine functional group.

### Binding Energy and Bader Charge Analysis

In order to provide a more quantitative analysis of water and hydroxide interactions, the binding energy of the monomer–water complexes is calculated for the different structures. The binding energies (*E*_b_) were computed from the energies of the relaxed structures, obtained after the first structure optimization using density functional theory with GGA-PBE exchange correlation and Van der Waals interactions added by means of the DFT + D2 approach of Grimme. They were calculated using eq 1
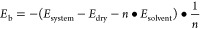
1where *n* is the number of solvent molecules (water molecules or hydroxide ions) in the system, *E*_system_ and *E*_dry_ are the energies of the hydrated and dry monomer, respectively (in NH_2_ or N(CH_3_)_3_ form), as obtained at the termination of VASP structure optimization, and *E*_solvent_ is the energy of a single gas-phase solvent molecule in a vacuum. The latter was calculated by placing a single molecule in a spacious vacuum (10 × 10 × 10 Å^3^ simulation box) to avoid any interaction between periodic copies. The structure was subsequently optimized using the DFT GGA-PBE functional with Van der Waals corrections and accurate precision in reciprocal space.^52^ Each binding energy was calculated per solvent molecule.^53^ The obtained values are reported in Table 1.

**Table 1:** Binding Energy Values Calculated for Different Structures with the GGA-PBE Functional and DFT + D2 vdW

structure	*E*_dry_ [eV]	*E*_solvent_ [eV]	*E*_system_ [eV]	*N*	*E*_b_ [eV]	*E*_b_ [kJ/mol]
structure 1	–205.861	–14.212	–220.242	1	0.169	16.306
structure 2	–205.861	–14.212	–220.340	1	0.267	25.762
structure 4	–255.907	–7.158	–267.663	1	4.598	443.647
structure 5	–255.907	–7.158	–267.520	1	4.455	429.849
structure 6	–205.861	–7.158	–214.514	1	1.495	144.248
structure 7	–255.907	–11.861^a^	–297.809	3	2.107	203.266
structure 8	–255.907	–12.801^a^	–327.141	5	1.446	139.501

a Average values calculated considering both water molecules and hydroxide anions.

From the above results, it emerges that in neutral systems (structures 1–2), a single water molecule bounds more strongly to the carbonyl group, with an excess energy of 0.098 eV, with respect to the amine group, consistent with the previous observations. The final structure 4 showed an increase of *E*_b_ of 4.429 eV, with respect to structure 1. The much higher binding energy may be attributed to the proton transfer from the amine group to the hydroxide anion, while only formation of a hydrogen bond or weak interaction is observed in the first two structures. However, from DFT structure optimization, we find that the interaction between the new water molecule and the head group is weaker (no hydrogen bond formation). This result can be proved by means of the charge redistribution analysis discussed below. The binding energy of structure 5 was very close to that of structure 4 (only 0.143 eV difference) due to the same final interaction, whereas structure 6 showed a higher binding energy with respect to neutral systems, ascribed to the dissociation of NH_2_ into the NH group, but still lower than structures 4 and 5.

Finally, looking at the energy values of structures 7 and structure 8, we conclude that an increase in water coverage reduces the binding energy. At these levels of hydration, the formation of water clusters with hydrogen bonds among water molecules is predominant, and it is shielding the interaction with the polymer.^53^ This shielding effect is beneficial for enhancing ionic conductivity since less energy is required for the hydroxide anion to diffuse throughout the membrane.

For comparison, the same analysis was executed using Van der Waals density functional optPBE-vdW.^39,54^ Although the obtained values (Tables S3) for the binding energies were different, the general trend was the same, allowing for consistent conclusions to be drawn.

A further verification can be provided by the determination of atomic charges. A correlation exists between binding energies and charge transfer: stronger water–monomer interactions are accompanied by larger charge redistributions.^55^ The net charge transfer can be calculated as the difference between the partial charge of the single interacting water molecule before and after structure optimization, considering that it defines the total charge that is exchanged with the region of interest of the monomer.

The calculation of atomic partial charges and charge transfer was performed by means of Bader charge analysis.^38^ These were estimated for initial and final configurations of structures 1, 2, 4, 5, 7, and 8, with particular regard to the amine head groups and water molecules. The obtained values of net Bader charges and charge transfer to/from the water molecule are reported in Table 2.

**Table 2:** Net Bader Charges of Interacting Groups and Charge Transfer in the Water Molecule in Units of the Electronic Charge (e)

structure	net bader charge of amine group	net bader charge of carbonyl group	charge transfer
structure 1	–0.35		2 × 10^–6^
structure 2	–0.33	–1.07	–0.003
structure 4	–0.22		0.470
structure 5	–0.25	–1.12	0.650
structure 7	0.48		–0.028
structure 8	0.47		–0.037

A stronger interaction was obtained in structure 4 and 5, exhibiting a larger charge redistribution compared to neutral systems. Moreover, larger electronegativity of the interacting region ensures a stronger electrostatic interaction with the hydrogen atom of the water molecule, while repelling the hydroxide anions. The deprotonated TMA group in the final configuration of structure 4 was found to be slightly negative, in opposition to the starting cationic group, thus enhancing the electrostatic interaction with the produced water molecule compared to the positive pendant chain.^56^ However, the lower negative charge, relative to structure 1 and 2, is a proof of the weaker interaction with the new H_2_O molecule, which may be attributed to Van der Waals type dispersion forces only. The very strong negative character of the carbonyl group, on the other side, is found to enhance water adsorption (structure 2), while strongly repelling the hydroxide ion (structure 5), which is instead attracted by the positive charge of the TMA. Finally, the hydrated systems (structures 7 and 8) both retain a partial positive charge on the amine group, which points out the lack of interaction with the anionic species and degradation. The relatively high charge redistribution of the OH^–^ might be instead attributed to the interaction with the water molecules in the solvation shell.

### Chemical Degradation and Activation Energy

To investigate the degradation reaction of the TMA head group at different levels of hydration, the reaction energy (Δ*E*_R_) and the activation energy (Δ*E*_A_) were estimated via DFT calculations of the energies of transition state geometries.^47,49,50^

The transition state geometries were selected from the trajectory of structure 4 in gas-phase conditions, therefore, considering only the Ylide formation mechanism. The hydrated conditions were simulated applying the implicit solvation model instead of explicitly introducing water molecules, to reduce the computational cost.^47−49^ The geometries of the transition states were kept fixed, only varying the dielectric constant ε, set equal to 78.4, to simulate high water content and equal to 40 for intermediate hydration. However, it is important to point out that the implicit solvation model only approximately accounts for the presence of a dielectric constant, not considering the chemical interaction with water molecules.

For all the transition geometries, the energies were calculated based on the hybrid functional B3LYP with Grimme Van der Waals corrections. Subsequently, Δ*E*_R_ and Δ*E*_A_ were computed according to the eqs 2 and 3.^47^
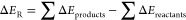
2
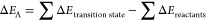
3

**Figure 10:**
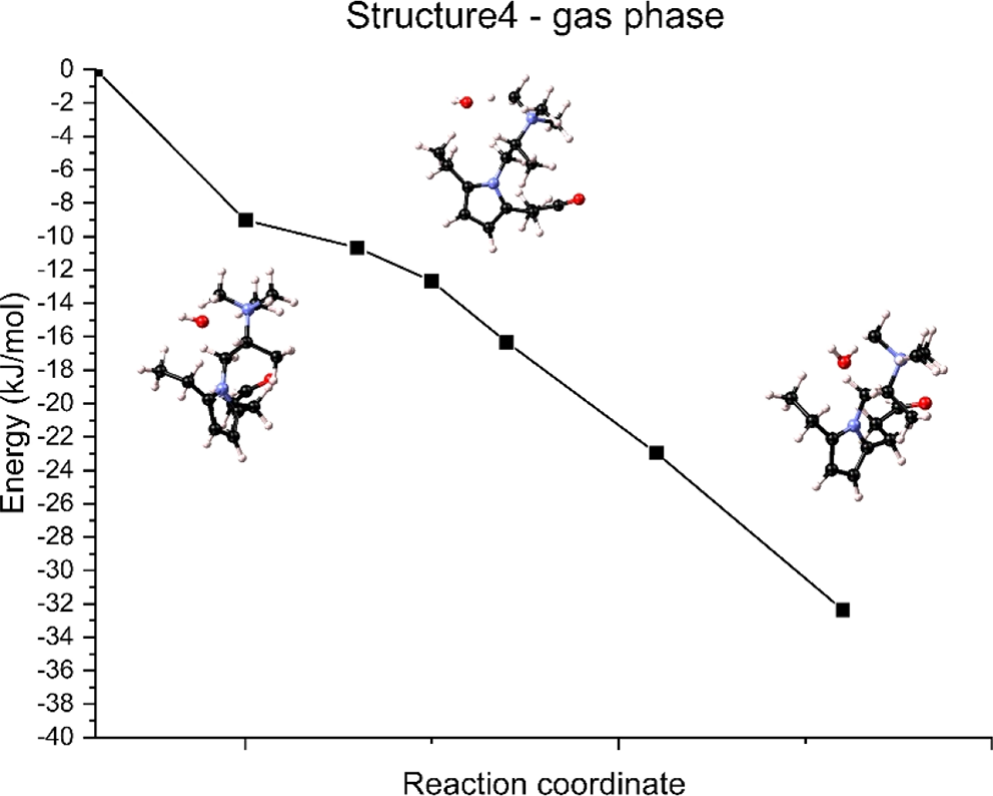
Degradation path via the Ylide formation mechanism of TMA in structure 4 in gas-phase conditions (ε = 0).

For structure 4 in dry conditions (ε = 0), the degradation reaction mechanism did not show any energy barrier, underscoring the strong reactivity of the anionic species in the absence of the shielding effect of water molecules (Figure 10). The zero-energy condition was selected as one of the first transition state. The calculated reaction energy was Δ*E*_R_ = −32.35 kJ/mol, thus indicating the exothermic nature of the reaction.

**Figure 11:**
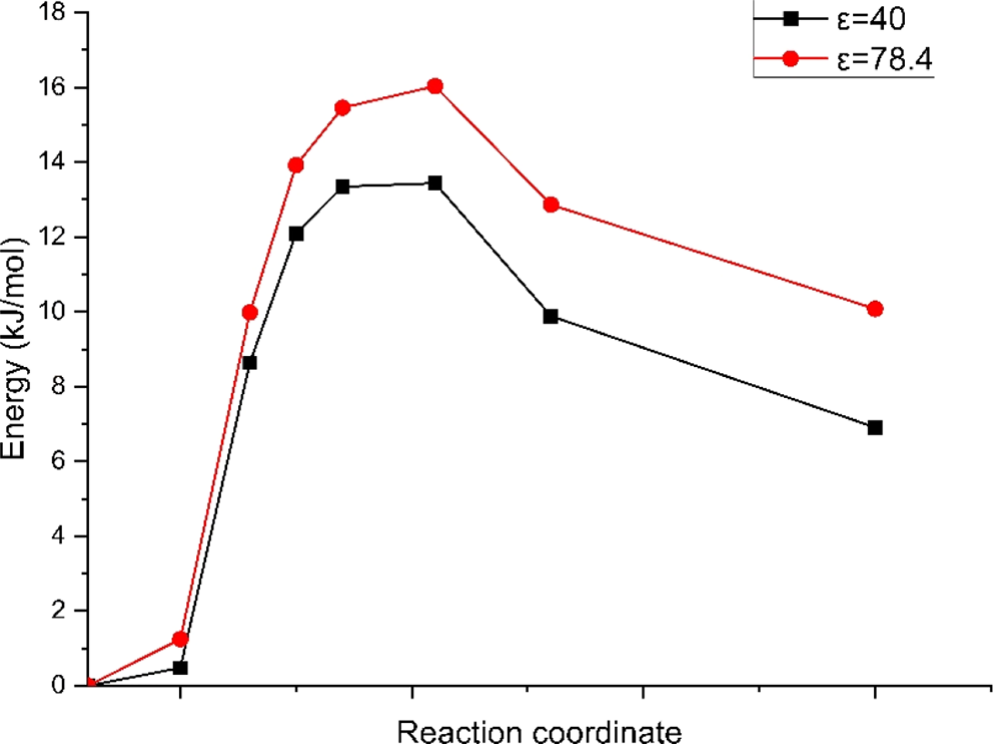
Degradation path via the Ylide formation mechanism of TMA in structure 4 at intermediate hydration conditions (ε = 40) and high hydration conditions (ε = 78.4).

Medium (ε = 40) and high (ε = 78.4) hydrations both presented an energy barrier, with activation energies equal to 13.44 and 16.03 kJ/mol, respectively. The respective reaction energies were 6.91 and 10.08 kJ/mol, the positive values indicating an endothermic reaction (Figure 11).

The computed energies are low compared to other studies,^47−51^ sign of lower chemical stability of the monomer. However, it should be noted that the implicit solvation model significantly underestimates the obtained values which would be higher, considering explicit water molecules.

The increment in Δ*E*_A_ and Δ*E*_R_ with an increase of the hydration level confirms once again the important role of water molecules in preserving the cationic group from OH^–^ attack and alkaline degradation.

## Conclusions

In the present article, the structural properties of the various functional groups in polyamine, namely, the carbonyl group, the pyrrolic ring, and the amine group (both ionized and non-ionized), and their interaction with water molecules and hydroxide anions were investigated by means of DFT. Simulation results highlight the role of water molecules and hydroxide anions in the formation of water clusters within the membrane and in the transport of anionic species. Structure relaxations, followed by ELF mapping and Bader charge analysis, were used to identify the nature of the bonding. The carbonyl group and the amine functional group displayed a hydrophilic behavior, whereas the backbone and the pyrrolic ring are hydrophobic.

The carbonyl group has a role in water uptake since it shows the strongest interaction with water molecules in terms of the hydrogen bond, but it does not have any active role in ionic conductivity. On the other hand, methylated amine groups play the leading role in ionic diffusion, but at lower hydration levels, are strongly subjected to chemical degradation via Ylide formation reactions.

An increase of the water content is essential for enhancing conductivity and preventing chemical degradation owing to a reduced interaction with hydroxide anions (decrease in the binding energy and increase in the activation energy) upon the shielding effect of water molecules that tends to aggregate forming water clusters in the proximity of the TMA and solvating the anionic species.

The formation of this hydrogen-bonded cluster points to the strong involvement of the N(CH_3_)_3_^+^ functional groups in anion transport at high levels of hydration, providing preferential pathways for ionic conductivity.

## Supporting Information Available

Distances and angles involving N1 and N2 atoms, optimized structures and N pair correlations of structures 1–8, and binding energy values calculated with optPBE-vdW functionals (PDF)
Animated pathway during structure optimization of structure 1. Animated pathway during structure optimization of structure 2. Animated pathway during structure optimization of structure 3. Animated pathway during structure optimization of structure 4. Animated pathway during structure optimization of structure 5. Animated pathway during structure optimization of structure 6. Animated pathway during structure optimization of structure 7. Animated pathway during structure optimization of structure 8 (ZIP)
