# Supplementary material for: Water Uptake in an Anion Exchange Membrane Based on Polyamine: A First-Principles Study
Source: J Phys Chem B. 2022 Sep 19;126(38):7418–28. doi: 10.1021/acs.jpcb.2c04115 (PMC9527750; doi:10.1021/acs.jpcb.2c04115)
Supplement: Supplementary file 1 — jp2c04115_si_001.pdf [file jp2c04115_si_001.pdf]

Supporting Information

Water Uptake in Anion Exchange Membrane based on Polyamine: A First Principles Study

Eleonora Tomasino, Binayak Mukherjee, Narges Ataollahi, Paolo Scardi*

Department of Civil, Environmental and Mechanical Engineering, University of Trento, Via Mesiano, 77, 38123, Trento, Italy

Table S1: Distances and angles involving N1 atom

N1	*Distance (Å)*			*Angles*		
Monomer	**N1-C2**	**N1-C7**	**N1-C4**	**C4-N1-C2**	**C7-N1-C2**	**C7-N1-C4**
1	1.377	1.415	1.421	112.733	123.936	123.329
2	1.412	1.455	1.482	106.063	125.958	121.350
3	1.342	1.471	1.418	112.121	124.793	122.964
4	1.386	1.490	1.462	110.301	126.670	123.024
5	1.384	1.543	1.434	110.412	126.035	123.096
6	1.371	1.464	1.399	110.412	132.009	117.517
7	1.341	1.468	1.400	109.818	125.435	123.406
8	1.394	1.467	1.438	108.939	125.259	125.797

9	1.365	1.469	1.377	110.079	122.730	126.737
10	1.391	1.458	1.424	113.394	123.135	122.562
Average	**1.376**	**1.470**	**1.425**	**110.427**	**125.596**	**122.978**

Table S2: Distances and angles involving N2 atom

N2	*Distance (Å)*			*Angles*		
Monomer	**N2-H14**	**N2-H13**	**N2-C11**	**H13-N2-C11**	**H14-N2-C11**	**H14-N2-H13**
1	1.042	1.024	1.474	105.910	109.712	98.937
2	1.029	0.990	1.495	105.352	107.060	110.701
3	1.041	1.024	1.439	108.264	121.006	115.426
4	1.035	0.960	1.442	109.290	106.522	103.019
5	1.048	0.996	1.495	102.442	104.550	104.790
6	1.058	0.998	1.430	114.524	106.544	100.810
7	1.024	1.012	1.414	112.445	111.201	99.820
8	1.028	0.987	1.424	114.691	99.245	100.751
9	1.102	1.040	1.540	114.580	106.720	105.243
10	1.029	1.003	1.429	112.798	110.793	106.941
Average	**1.044**	**1.003**	**1.458**	**110.030**	**108.335**	**104.644**

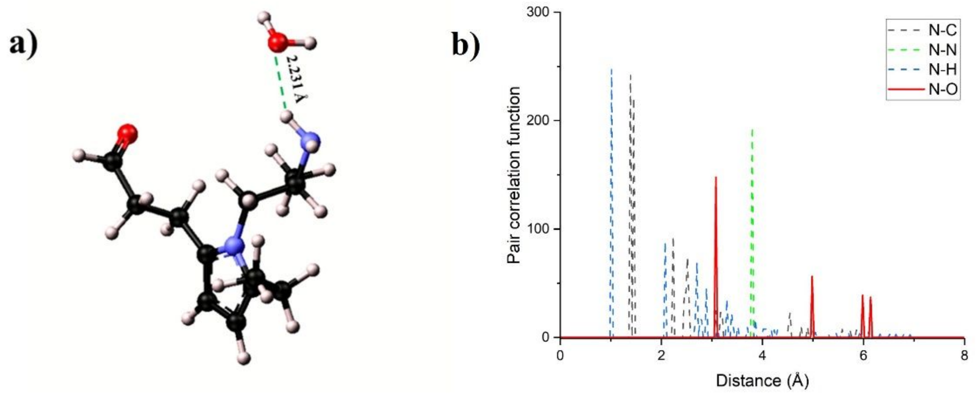

Figure S1: Optimized structure (a) and N pair correlation (b) of Structure 1

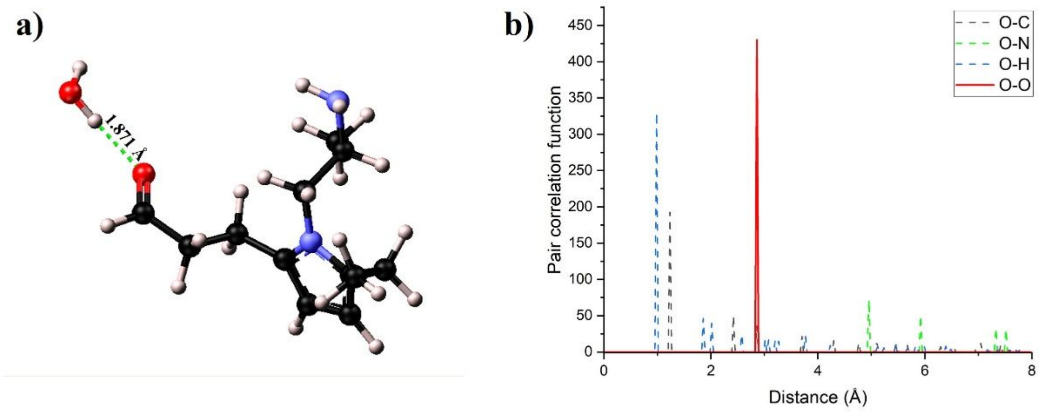

Figure S2: Optimized structure (a) and O pair correlation (b) of Structure 2.

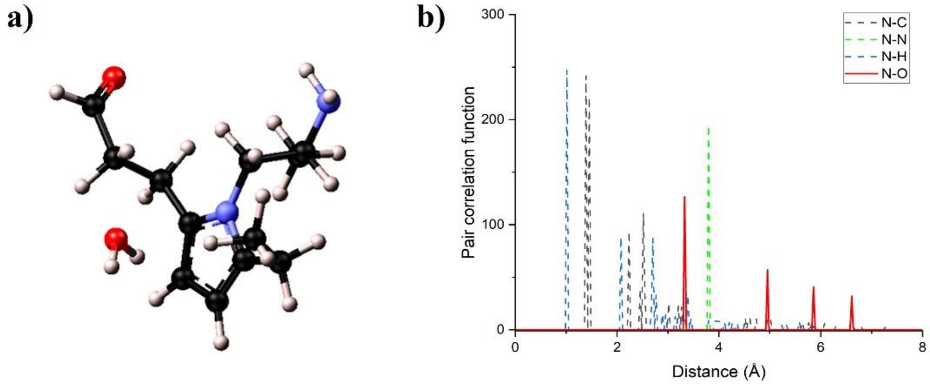

Figure S3: Optimized structure (a) and N pair correlation (b) of Structure 3.

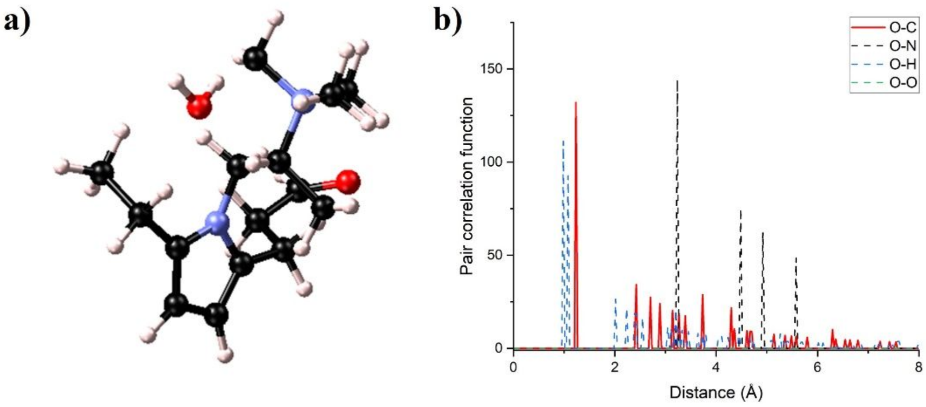

Figure S4: Optimized structure (a) and O pair correlation (b) of Structure 4.

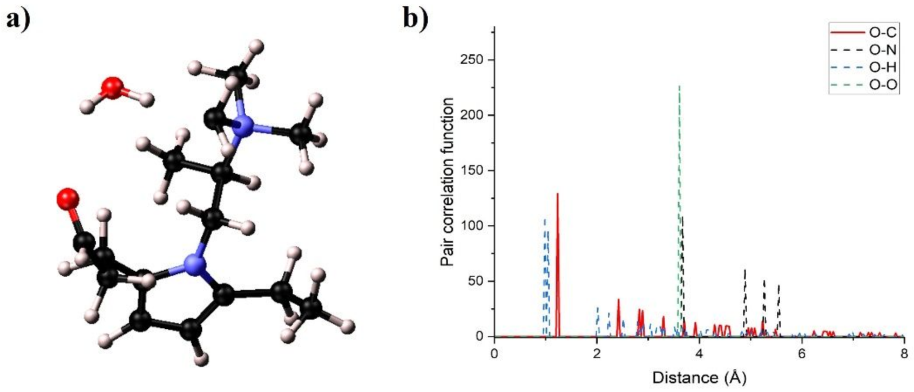

Figure S5: Optimized structure (a) and O pair correlation (b) of Structure 5.

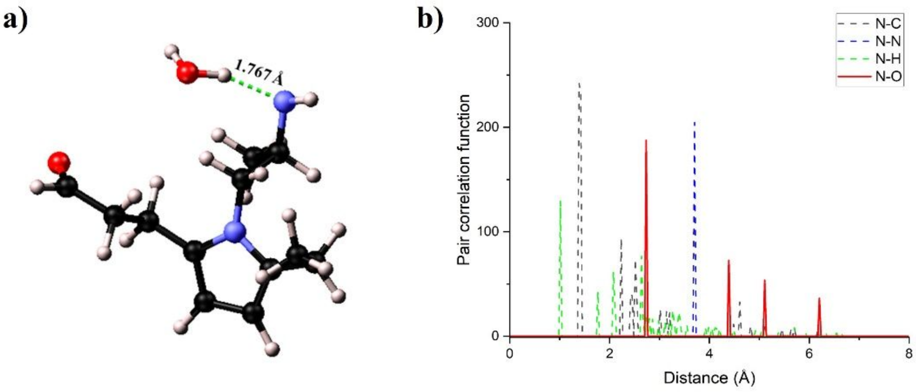

Figure S6: Optimized structure (a) and N pair correlation (b) of Structure 6.

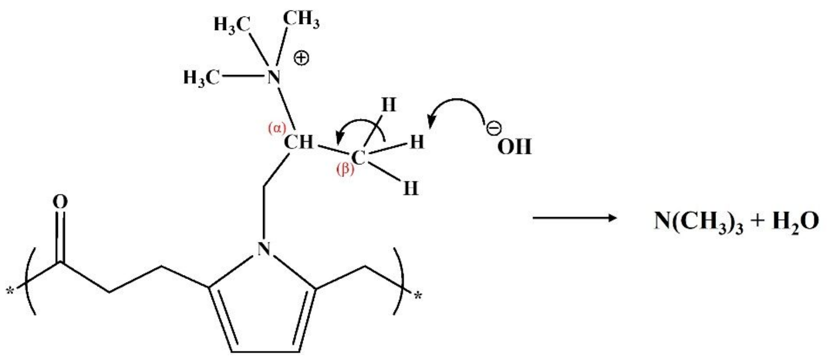

Scheme S1: Hofmann elimination reaction

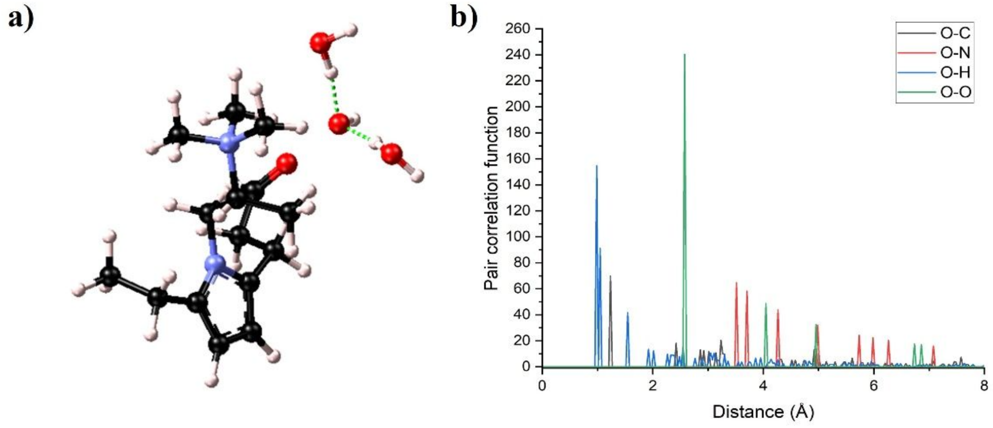

Figure S7: Optimized structure (a) and O pair correlation (b) of Structure 7.

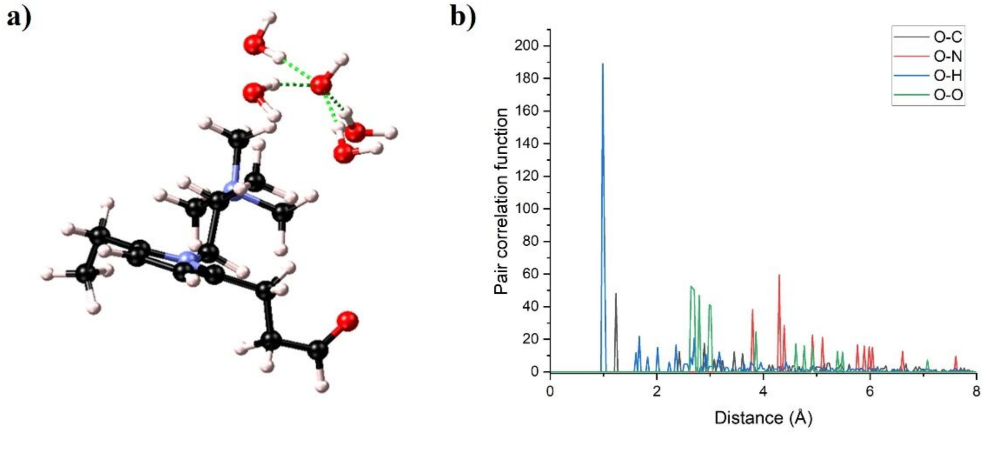

Figure S8: Optimized structure (a) and O pair correlation (b) of Structure 8.

Table S3: Binding energy values calculated for different structures with optPBE-vdW functional

Structure	E_{dry} [eV]	$E_{solvent}$ [eV]	E_{system} [eV]	n	E_b [eV]	E_b [kJ/mol]
**Structure 1**	-181.938	-12.200	-194.300	1	0.162	15.631
**Structure 2**	-181.938	-12.200	-194.389	1	0.251	24.218
**Structure 4**	-227.269	-5.287	-237.092	1	4.536	437.665
**Structure 5**	-227.269	-5.287	-236.774	1	4.218	406.982
**Structure 6**	-181.938	-5.287	-188.622	1	1.397	134.792
**Structure 7**	-227.269	-9.896*	-262.603	3	1.882	181.621
**Structure 8**	-227.269	-10.817*	-288.010	5	1.331	128.405

(* average values calculated considering both water molecules and hydroxide anions)
